# Prevalence and associated factors of Depression and Post Traumatic Stress Disorder (PTSD) among internally displaced children in Nigeria

**DOI:** 10.4314/ahs.v25i4.21

**Published:** 2025-12

**Authors:** Chidiogo L Umennuihe, Franca O Okechukwu, Uju I Nnubia, Ezinne J Nwauzoije

**Affiliations:** Department of Home Science and Management, University of Nigeria, Nsukka

**Keywords:** Mental health, Children, Depression, Internal displacement, Posttraumatic stress disorder

## Abstract

**Background:**

Internally displaced children are at risk of not receiving the general health services they require, since they have health needs that are unique to those of adults. Therefore, the first step in designing intervention programs for them is to determine their mental health status.

**Objectives:**

This study determined the relationship of sociodemographic factors with the prevalence of depression and PTSD among internally displaced children in Nigeria.

**Methods:**

A descriptive correlational research design was adopted for the study using adapted versions of the Child PTSD Symptom Scale (CPSS) and the child version of the Revised Child Anxiety and Depression Scale – RCADS (Depression Subscale). Frequency, percentage, binary logistic regression and chi-square were used for data analysis.

**Results:**

Findings showed that the participants (n=474) comprised 55.3% male and 44.7% female children, with 85.9% having a low household income. The prevalence rate of PTSD and depression among displaced children was 79.7% and 84.8%, respectively. More than a third (47.5%) of the children were severely depressed, with more male (28.2%) than female children (21.2%) having moderately severe depression. A greater proportion of male (39.3%) than female (30.7%) children had severe PTSD. Functional impairment due to PTSD was found in 90.7% of the children. At a 95% confidence interval, age and gender did not significantly predict the prevalence of depression and PTSD among internally displaced children in Nigeria (OR < 1 and P > 0.05). At p<0.05, demographic characteristics of the children, such as parents' education, household size and housing size, correlated significantly with the prevalence of PTSD and depression among them.

**Conclusions:**

Findings of this study suggest a high prevalence of depression and PTSD among displaced children in Nigeria and therefore call for the government to make available counselling and rehabilitation services to all IDPs, especially the children, to improve their mental health.

## Introduction

The psychological well-being of children is often overlooked despite the fact that they experience mental health issues such as depression and PTSD. According to Chinawa et al.^1^, it is well recognised and established that children and adolescents suffer from these problems. However, it is frequently attributed to the usual stress experienced at this age. The World Health Organisation estimates that as many as 20% of the world's children and adolescents experience a mental health problem at some stage in their childhood^2^. Neglecting mental health concerns in children and adolescents can have grave and detrimental effects, as they may end up struggling with psychological issues throughout their lives^20^. Chinawa et al.^3^ asserted that resilience and social support can be effective in managing the symptoms of poor mental health in children and adolescents. In the absence of these, children and adolescents may resort to negative coping strategies (NCS) such as disengagement, denial, blame, and social withdrawal to alleviate their poor mental health, which can exacerbate the symptoms^4^. This is particularly true for internally displaced persons (IDPs), who have been compelled to leave their places of habitual residence as a result of armed conflicts, widespread violence, human rights violations, natural or man-made disasters, and have not crossed an internationally recognised state border^5^. According to Sambo^6^, these IDPs live with pains and memories that may never make them the same again. They are therefore among the world's most vulnerable populations^7^.

Globally, the overall displacement rate has continued to increase, with Asia and Africa accommodating a larger proportion of the displaced population^8^. According to the Africa Centre for Strategic Studies, over 25 million internally displaced persons can be found in Africa^9^. As of February 2023, 2,375,661 IDPs were identified in Northeast Nigeria, while up to 1,087,875 persons have been displaced in the Middle Belt region of Nigeria^10^. This high number is a result of Nigeria's ongoing diverse array of security challenges, most notably violent extremism in the northeast^2^ and attacks by Fulani herders in northcentral Nigeria. These conflicts cut across religious differences, tensions over resources and land, worsened by climate change, and have displaced a significant number of people^11^, 20 per cent of whom are living in camps or camplike settings, while 80 per cent are in host communities^10^. These traumatic experiences inevitably present a plethora of physical and psychological obstacles to the affected population^12^.

Due to loss of lives and properties, insecure living conditions, overcrowding, poverty, food insecurity, unemployment, discrimination in aid distribution, and sexual and gender-based violence^12^, IDPs have a greater risk of developing mental health problems. Several studies^5^,^13^,^14^ reported that post-traumatic stress disorders (PTSD) in response to violence or severe traumatic life stress, as well as depression in response to loss, are the most commonly reported mental health issues among IDPs. Post-traumatic stress disorder (PTSD) is a psychological condition that arises when someone experiences or witnesses a stressful life event such as abuse, natural disaster, violence and warfare, which jeopardises their mental and physical well-being^15^. Depression, on the other hand, is an emotional condition that results in a continuous sense of sadness and a loss of interest in one's regular activities^1^. The emotional trauma and behavioural changes associated with these mental illnesses can strain family relationships, causing conflict, neglect, and additional emotional pain. They can also cause secondary issues like substance abuse, self-harm, and suicide^16^,^1^,^17^, which have increased three times in the past 50 years among children and adolescents^1^.

The mental health of internally displaced children is of special concern due to their early experiences with insecurity, socio-economic deprivation and violence^18^. However, an extensive literature search showed no documented studies that assessed the severity of PTSD and depression in displaced children in Plateau State, Nigeria. Most of the studies on the mental health of IDPs in Nigeria and Africa have focused solely on adults, neglecting the particularly vulnerable nature of children. For instance, Gebreyesus et al.^19^ found an 81.2% prevalence of depresion among the displaced population in Tigray, Ethiopia, with more than 60% classified as moderate to severe depression. Similarly, Madaro et al.^20^ estimated that 58.4% of the displaced population in South Ethiopia suffered from PTSD. Additionally, Mohamed and Kheir^13^ in their study reported that based on DSM-IV symptoms' criteria, the prevalence of PTSD and depression among IDPs in Sudan was 25% and 62%, respectively. A study by Olufadewa et al.^14^ showed that 54.6% of IDPs in Northern Nigeria met the diagnostic criteria for depression, while 19.9% had current PTSD symptoms. This puts the prevalence of depression and PTSD among the adult displaced population between 19% and 81%. To the best of the researchers' knowledge, this is the first study to evaluate the mental health of internally displaced children in Nigeria and Plateau State.

The presence of mental health issues may exacerbate difficulty in adjusting to everyday life after relocation. As such, widespread depression, feelings of fatigue and apathy may discourage IDPs from taking steps to improve their situation^21^. Although adults are becoming more aware of their mental health, the same cannot be said for children^17^, and even less so for internally displaced children. Again, in Nigeria, there is a dearth of mental health interventions available for children affected by conflict; in fact, treatment gaps for children are much greater than those for adults^22^. This study is therefore essential as it aimed to assess the prevalence and severity of PTSD and depression, as well as determine the relationship of demographic factors with these mental health problems among internally displaced children in Nigeria. This will help in assessing the burden of mental health illnesses among this vulnerable group, allowing recommendations to be made for specific types of interventions that would improve the overall mental health status of the displaced population.

## Methods

### Study area

This cross-sectional study was conducted between December 2019 and January 2020 in IDPs' camps in Plateau State, Nigeria. Plateau State has been a venue of clashes between the predominantly Muslim Hausa-Fulani herders and Christian farmers, like the other states of the Middle Belt region in Nigeria^23^.

### Study population

Due to security reasons, two IDPs' camps in Plateau State were conveniently selected for the study, and the population of 6 – 12-year-old children in these two camps at the time of the study was 94,8^24^.

### Sampling technique

To determine the sample size for the study, 50% of the children's population was calculated, resulting in a sample size of 474.

A simple random sampling technique was used to select the required number of children from each camp, allowing them an equal chance of being selected.

### Study design

A descriptive correlational research design was adopted for this study. The design was the most suitable because this study aimed to describe the relationship between the mental health status (prevalence of depression and PTSD) and sociodemographic characteristics of displaced children.

### Inclusion criteria

Children aged 6 – 12 years who have been displaced for at least a month and live in a household with parents or adult guardians.

### Exclusion criteria

Children who have been displaced for less than one month.

### Research instruments

Socio-demographic characteristics questionnaire: This was designed by the authors to elicit information on the respondents' sociodemographic characteristics such as age, gender, household income and size, and parents' education.

Child version of the Revised Child Anxiety and Depression Scale – RCADS (Depression Subscale) by Chorpita, Moffitt and Gray^25^: The RCADS – Depression subscale is a 10-item questionnaire that measures the reported severity of various symptoms of depression. Each item asked the child to rate the severity of their depression symptoms during the past 7 days on a scale of 0-3. The total score ranged from 0 to 30, with higher scores indicating greater severity of depression.

Child PTSD Symptom Scale (CPSS) by Foa, Johnson and Feeny^26^: The CPSS was used to assess the severity of post-traumatic stress disorder in children. The questionnaire consisted of 17 items in part 1 (severity of PTSD) and seven items in part 2 (Functional impairment due to PTSD). For scoring the severity of PTSD symptoms, each of the first 17 items was rated on a scale from 0 to 3, with a total score ranging from 0 to 51. In the second part, participants were asked how much the problems indicated in section one interfered with specific areas of life. These seven questions were scored dichotomously as absent (0) or present (1). Scores ranged from 0 to 7, with higher scores indicating greater functional impairment.

### Validation and reliability of the instruments

Face and content validation of the socio-demographic characteristics questionnaire was conducted by three experts from the field of family and child studies. A Cronbach's alpha coefficient of 0.85 was obtained for the instrument, indicating a good internal consistency.

Since no record of the use of the CPSS was found in Nigeria, it was taken for face and content validation by three experts from the field of Psychology. A Cronbach's alpha coefficient of 0.71 was obtained for the instrument, indicating an acceptable internal consistency.

The RCADS was validated and evaluated for reliability during a cross-cultural study that included Nigeria, and it obtained a very acceptable reliability coefficient (Cronbach's alpha) of 0.91^27^

### Data collection methods

Data was collected using interviewer-administered questionnaires. Three research assistants were recruited and trained to administer the questionnaires to the respondents. The research assistants were required to be fluent in English and Hausa to interpret the questionnaire items easily for the subjects. The questionnaires were administered to children who met the inclusion criteria together with their parents or adult guardians.

### Data analysis and management

The data collected were coded into IBM-SPSS, version 23, for analysis. Household monthly income was classified as low (Less than ₦ 10,000), moderate (₦ 10,000 - ₦ 30,000) and high (₦ 40,000 - ₦ 60,000). To obtain the prevalence of depression, children with a score of 10 or above on the RCADS (Depression subscale) were categorised as having depression, while those with a score below 20 were classified as having no depression. For severity of depression, scores of 0-4 showed absence of depression; 5-9 – Mild depression; 10-14 – moderate depression; 15-20 – moderately severe depression; and 21-30 – severe depression. To obtain the prevalence of PTSD, children with a score of 20 and above on the CPSS were categorised as having PTSD, while those with a score below 20 had no disorder. For severity of PTSD, scores of 0-10 were regarded as below threshold; 11-15 – subclinical; 16-20 – mild PTSD; 21-25 – moderate PTSD; 26-30 – moderately severe PTSD; 31-40 – severe PTSD; and 41-51 - extremely severe PTSD. Frequencies and percentages were used to analyse data on all variables. The relationship between categorical variables was compared using the chi-square test. A binary logistic regression, with its odds ratio at a 95% confidence interval, was used to determine age and gender as predictors of depression and PTSD. The significance was set at a p < 0.05 level of significance. Tables, pie charts, and bar charts were used to present the data.

## Ethical considerations and informed consent

Ethical approval was obtained from the Ethics and Research Committee of the institution close to the study area, Jos University Teaching Hospital (JUTH), Jos, Plateau State. The ethical approval certificate had the reference number JUTH/DCS/IREC/127/XXX/2162. Before the commencement of data collection, oral and written consent was obtained from the parents/guardians of the children, and assent was obtained from the children.

## Results

Data was collected from 474 eligible participants. According to Table 1, the respondents' gender distribution showed that 55.3% were males while 44.7% were females. More than half (51.3%) were aged 6-9 years. Primary education (43%) was the most common education level of the parents, and farming (52.5%) was the primary source of income. Up to 85.9% recorded low household monthly income; the most common household size was 5-8 (71.5%). Most (87.6%) of the children and their families have been displaced for over twelve months.

**Table 1:** Sociodemographic characteristics of the children

Variables	Frequency	Percentage
**Gender**		
Male	262	55.3
Female	212	44.7
**Age (years)**		
6-9	243	51.3
>9-12	231	48.7
**Education level of parent/guardian**		
No formal education	164	34.6
Primary education	204	43.0
Secondary education	106	22.4
**Source of income for the household**		
Farming	249	52.5
Business/Self-employment	29	6.1
Support from the government and charity organisations	196	41.4
**Household monthly income**		
Low	407	85.9
Moderate	60	12.7
High	7	1.4
**Household size**		
Less than five	6	1.3
Five to eight	339	71.5
More than eight	129	27.2
**Duration of displacement**		
Twelve months or less	59	12.4
More than twelve months	415	87.6

**Figure 1:**
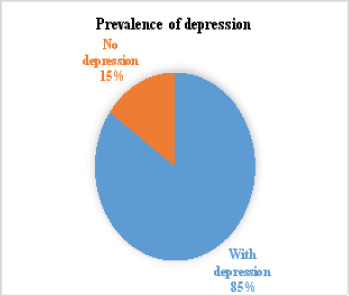
Prevalence of depression

**Figure 2:**
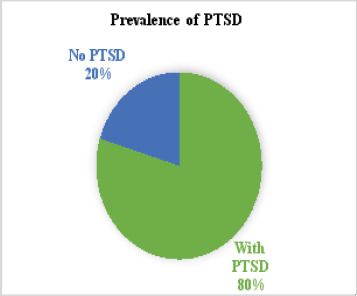
Prevalence of PTSD

The prevalence rate of depression and PTSD among displaced children in Nigeria was 79.7% and 84.8%, respectively (Figures 1 and 2).

**Figure 3:**
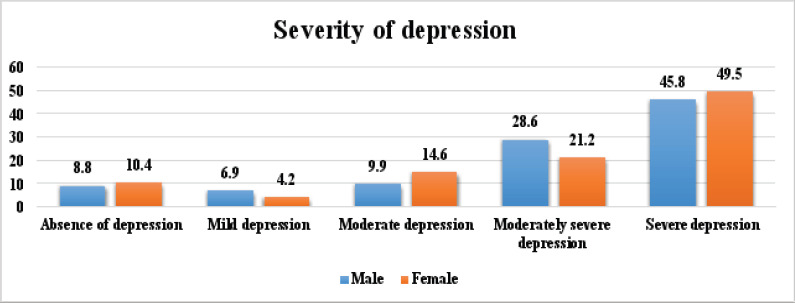
Severity of depression according to gender

According to Figure 3, more female (49.50%) than male children (45.8%) had severe depression, while more male (28.6%) than female (21.2%) children had moderately severe depression.

**Figure 4:**
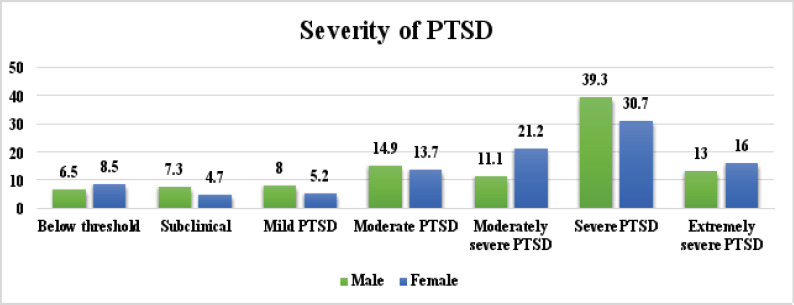
Severity of PTSD based on gender

More male (39.3%) than female children (30.7%) had severe PTSD, while more female (16%) than male children (13%) had extremely severe PTSD (Figure 4).

**Figure 5:**
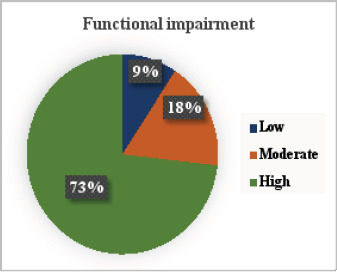
Functional impairment due to PTSD

According to Figure 5, 73% of the displaced children experienced high functional impairment due to PTSD, while only 9% did not experience it.

Table 2 shows that age and gender do not significantly predict the prevalence of depression and PTSD among internally displaced children in Nigeria (OR < 1 and P > 0.05). The odds ratio of depression for male children compared to female children is 0.923, while the odds ratio of PTSD is 0.999. The odds ratio of depression for older children (>9-12) compared to younger children (6-9) is 0.823, while the odds ratio of PTSD is 0.787.

**Table 2:** Gender and age as predictors of depression and PTSD

Variables	Odds Ratio	P	95% Confidence Interval	
			Lower	Upper
**Depression**				
Gender	0.923	0.758	0.556	1.533
Age	0.999	0.998	0.604	1.653
**PTSD**				
Gender	0.823	0.403	0.522	1.299
Age	0.787	0.300	0.501	1.237

Regarding the sociodemographic factors associated with the prevalence of depression among the children, chi-square analysis showed that parents' education (p=0.020), household size (p=0.001) and accommodation size (p=0.001) were significantly associated with the prevalence of depression. Depression was recorded more in children whose parents have primary education (88.2%) compared to other children. More children with a household size of more than eight (93%) had depression when compared to other children from smaller family sizes. The proportion of children with depression who had minimal accommodation (93.9%) was higher when compared to those with small and moderate-sized housing (Table 3). Although the children's education was not significantly correlated with the prevalence of depression among them, the proportion of those with depression (90.7%) who were not attending primary school was higher when compared to those attending primary school.

**Table 3:** Sociodemographic factors associated with the prevalence of depression and PTSD

	Depression		Posttraumatic stress disorder	
Demographic factors	No depression F (%)	With depression F (%)	No PTSD F (%)	With PTSD F (%)
**Gender**				
Female	31 (14.6)	181 (85.4)	39 (18.4)	173 (81.6)
Male	41 (15.6)	221 (84.4)	57 (21.8)	205 (78.2)
	***χ*^2^ = 0.96, df**			
	***χ*^2^ = 0.819, df = 1, p = 0.366**			
**Age (years)**				
6 – 9	37 (15.2)	206 (84.8)	54 (22.2)	189 (77.8)
>9 – 12	35 (15.2)	196 (84.8)	42 (18.2)	189 (81.8)
	***χ*^2^ = 0.001, df = 1, p = 0.982**		***χ*^2^ = 1.197, df = 1, p = 0.274**	
**Attending primary school**				
				
Yes	62 (16.9)	305 (83.1)	77 (21.0)	290 (79.0)
No	10 (9.3)	97 (90.7)	19 (17.8)	88 (82.2)
	***χ*^2^ = 3.664, df = 1, p = 0.056**		***χ*^2^ = 0.533, df = 1, p = 0.465**	
**Parents'/guardians' education level**				
			
No formal education	23 (14.0)	141 (86.0)	32 (19.5)	132 (80.5)
Primary education	24 (11.8)	180 (88.2)	32 (15.7)	172 (84.3)
Secondary education	25 (23.6)	81 (76.4)	32 (30.2)	74 (69.8)
	*χ*^2^ = 7.830, df = 2, p = 0.020*		*χ*^2^ = 9.169, df = 2, p = 0.010*	
**Source of income**				
Farming	40 (16.1)	209 (83.9)	56 (22.5)	193 (77.5)
Self-employment	2 (6.9)	27 (93.1)	2 (6.9)	27 (93.1)
Support from Government and NGOs	30 (15.3)	166 (84.7)	38 (19.4)	158 (80.6)
	***χ*^2^ = 1.698, df = 2, p = 0.428**		***χ*^2^ = 4.065, df = 2, p = 0.131**	
**Household monthly income**				
Low income	63 (15.5)	344 (84.5)	82 (20.1)	325 (79.9)
Moderate income	9 (15.0)	51 (85.0)	13 (21.7)	47 (78.3)
High income	0 (0.0)	7 (100.0)	1 (14.3)	6 (85.7)
	***χ*^2^ = 1.282, df = 2, p = 0.527**		***χ*^2^ = 0.231, df = 2, p = 0.891**	
**Household size**				
Less than five	3 (50.0)	3 (50.0)	3 (50.0)	3 (50.0)
Five to eight	60 (17.7)	279 (82.3)	75 (22.1)	264 (77.9)
More than eight	9 (7.0)	120 (93.0)	18 (14.0)	111 (86.0)
	*χ*^2^ = 14.055, df = 2, p = 0.001*		*χ*^2^ = 7.191, df = 2, p = 0.027*	
**Accommodation size**				
Very small	7 (6.1)	108 (93.9)	11 (9.6)	104 (90.4)
Small	54 (22.2)	189 (77.8)	66 (27.2)	177 (72.8)
Moderate	11 (9.5)	105 (90.5)	19 (16.4)	97 (83.6)
	*χ*^2^ = 19.658, df = 2, p = 0.001*		*χ*^2^ = 16.390, df = 2, p = 0.001*	
**Duration of displacement**				
Twelve months or less	6 (10.2)	53 (89.8)	8 (13.6)	51 (86.4)
More than twelve months	66 (15.9)	349 (84.1)	88 (21.2)	327 (78.8)
	***χ*^2^ = 1.318, df = 1, p = 0.251**		***χ*^2^ = 1.870, df = 1, p = 0.172**	

PTSD = posttraumatic stress disorder; F= frequency; % = percentage; *χ*^2^ = Chi-square value; p = Level of significance; df = degree of freedom

* Correlation is significant at p<0.05

From table 2, the sociodemographic factors that were significantly associated with the prevalence of PTSD among the children were parents' education (p=0.010), household size (p=0.027) and accommodation size (p=0.001). PTSD was recorded more in children whose parents have primary education (84.3%) compared to other children. More children with a household size of more than eight (86%) had PTSD when compared to other children from smaller family sizes. The proportion of children with PTSD who had minimal accommodation (90.4%) was higher when compared to those with small and moderate-sized housing. Although the children's education was not significantly correlated with the prevalence of PTSD among them, the proportion of those with PTSD (82.2%) who were not attending primary school was higher when compared to those attending primary school (Table 3).

## Discussion

This study determined the sociodemographic factors associated with the prevalence of depression and PTSD among internally displaced children in Nigeria. Displacement leaves negative socio-economic and health footprints on millions of people worldwide. From this study, the prevalence rate of depression and PTSD among internally displaced children in Nigeria was 79.7% and 84.8%, respectively, confirming the findings by Gebreyesus et al.^19^. This is probably because conflict and oppression, and subsequent forced displacement into unequipped and unsafe camps, predispose IDPs to mental health problems^28^. In this study, symptoms of depression, ranging from mild to severe depression, and symptoms of PTSD, ranging from mild to extremely severe, were found in over ninety per cent of the children. This suggests that an overwhelming number of children in IDPs' camps in Nigeria have become mentally unstable as a result of traumatic events and losses they have experienced in their young lives. This finding is comparable to that of Morgos et al.^29^, which showed that over 70% of displaced children in Southern Darfur met the DSM-IV criteria for PTSD, and more than one-third of them exhibited clinical symptoms of depression. Similarly, findings of studies^30^-^33^ all showed a high prevalence rate of mental health problems such as depression, PTSD and general distress among displaced adults and children in Iraq, Sudan and Uganda. Our finding, which showed that more female children had depression and PTSD compared to their male counterparts, is consistent with those of Hamida and Musa^34^ and Robert et al.^33^. It is also in line with the study of Tekin et al.^35^ in Turkey, which showed that more females than males suffered from PTSD and major depression. This finding also supports that of Taha and Sijbrandij^36^, which showed that female IDPs in Iraq reported more somatic and depressive symptoms than males. This high prevalence of PTSD and depression among displaced children, therefore, suggests that their current environmental and sociodemographic conditions are not optimal.

This present study showed that sociodemographic characteristics such as parents' education level, household size and housing size were significantly associated with the prevalence of depression and PTSD among the children. This finding contradicts that of AlShawi^31^, which showed a statistically insignificant association between the incidence of PTSD and gender, education level and monthly income of IDPs in Iraq. In this study, depression and PTSD were recorded more in children whose parents had primary education compared to those whose parents had no formal education or secondary education. The implication is that the lower the parents' academic level, the more the children may suffer from depression and PTSD symptoms. This may be because adults with higher educational qualifications or those who had obtained informal education may have more effective coping strategies for their wards who have experienced traumatic events and loss. Supporting this finding, a study by Alkhafaji et al.^30^ found that the level of education and income of IDPs in Iraq were significantly associated with the rate of depression among them.

Findings of this study suggest that a larger household size and very small-sized accommodations predispose internally displaced children to depressive and PTSD symptoms. This can be attributed to the tendency of larger household sizes to elevate stress levels in children, potentially negatively affecting their mental health, especially when combined with overcrowding. Furthermore, having more people to cater for in the face of uncertainty will exacerbate mental health problems in children, since most of their needs may be left unmet. Many people occupying a room, termed overcrowding, increases the risk of poor mental health^37^. Supporting this finding, a study by Pengcheng et al.^38^ found that household overcrowding is hazardous to mental health, emphasising the need for effective intervention strategies to alleviate the negative impacts of overcrowding on children's mental health. Similarly, Reed et al.^18^ stated that the combined weight of socioeconomic adversity, such as large family size, exposes children's mental well-being to several risks. According to Schupmann^39^, cramped and unsafe housing environments limit children's ability to explore, interact with, and learn from their surroundings. This can make children susceptible to feelings of extreme sadness and unhappiness. Our finding, however, contrasts with that of Grinde and Tambs^40^, who concluded that a large household is associated with fewer mental problems in children.

Although the children's education was not significantly correlated with the prevalence of depression among them, the proportion of children with depression and PTSD who were not attending primary school was higher when compared to those attending primary school. This was attributed to the fact that educational attainment is associated with better mental health^41^. According to Ahmad et al.^42^, internal displacement leads to mental health issues in children, which can be significantly reduced through education. Moreover, depriving internally displaced children of access to a good education can adversely impact families and society, perhaps resulting in persistent cognitive and mental health problems for these children^43^.

## Conclusion

The high prevalence of depression and PTSD in this study is comparable to other studies conducted among internally displaced persons. Although gender did not significantly predict the prevalence of depression and PTSD, more female than male children had depression and PTSD. Parents' education level, household size and housing size contributed substantially to the prevalence of depression and PTSD among the internally displaced children in Nigeria. The implication is that there is an urgent need for the government to make counselling and rehabilitation services available to all IDPs, especially the children, to improve their mental health.

### Study limitations

The vulnerable nature of the research participants was our major challenge in this study. Children require special protection from harm and exploitation, and their developing cognitive abilities may have affected their ability to recall information or provide reliable data. To mitigate some of these challenges, informed consent and assent were obtained from parents and children, respectively, after the study purpose and benefits had been explained to them. Another limitation is that the findings of this study may not be generalisable to other parts of the country where internally displaced persons are located. This highlights the need to research internally displaced children in different parts of the country experiencing insurgency. Additionally, there could be other factors, such as parents' mental health status, associated with the prevalence of depression and PTSD among the children that this study did not assess. Therefore, further research is needed to address other factors affecting the mental health of the displaced population.

## Data Availability

The data supporting the findings of this research are accessible from the corresponding author upon justified request.
